# The PopHEAD study: a population-based, cross-sectional study on headache burden in Norway: methods and validation of questionnaire-based diagnoses

**DOI:** 10.1186/s10194-024-01901-4

**Published:** 2024-11-20

**Authors:** Maria Bengtson Argren, Helene Engstrand, Andreas Kattem Husøy, John-Anker Zwart, Bendik Slagsvold Winsvold

**Affiliations:** 1 Department of Neurology, Oslo University Hospital, Oslo, Norway; 2 Norwegian Centre for Headache Research (NorHEAD), Norwegian University of Science and Technology, Trondheim, Norway; 3 Institute of Clinical Medicine, Faculty of Medicine, University of Oslo, Oslo, Norway; 4 Department of Research and Innovation, Division of Clinical Neuroscience, Oslo University Hospital, Oslo, Norway; 5 Department of Neurology and Clinical Neurophysiology, St. Olav University Hospital, Trondheim, Norway; 6 Department of Neuromedicine and Movement Science, Norwegian University of Science and Technology, Trondheim, Norway

**Keywords:** Headache burden, Epidemiology, Methodology, Migraine, Tension type headache, Medication overuse headache, HARDSHIP, PopHEAD

## Abstract

**Background:**

There is a lack of up-to-date information on the prevalence and burden of headache in Norway. Here we describe the methods and validation of the diagnostic tool of the PopHEAD study, a study designed to determine the prevalence and burden of migraine, tension-type headache, and medication-overuse headache.

**Method:**

PopHEAD is a Norwegian population-based cross-sectional study conducted in Vestfold and Telemark County in 2023. A random sample of 28,753 individuals aged 18 to 70 was invited to participate. The study used a digital version of the Headache-Attributed Restriction, Disability, Social Handicap and Impaired Participation (HARDSHIP) questionnaire, translated into Norwegian using the Lifting The Burden translation protocol. A subsample of participants was contacted by telephone within four weeks for an interview with a headache neurologist blinded to the questionnaire responses. Headache disorders were diagnosed according to the criteria of the International Classification of Headache Disorders version 3. Validity was expressed by sensitivity, specificity and Cohen’s kappa (κ).

**Results:**

In total, 8,265 (3,344 men and 4,921 women) responded. Most men (75.0%) and women (89.7%) reported having had a headache in the past year. Of 667 participants contacted for a telephone interview, 505 responded. The sensitivity and specificity of the questionnaire-based diagnoses were 97% and 72% for self-reported headache in the previous year (Cohen’s kappa κ = 0.72), 77% and 85% for migraine (κ = 0.61), 77% and 74% for tension-type headache (κ = 0.51), and 58% and 99% for medication-overuse headache (κ = 0.63), respectively.

**Conclusion:**

The PopHEAD questionnaire is a valid tool for identifying individuals with lifetime headache, migraine, tension-type headache, and medication overuse headache.

## Background

Headache disorders are increasingly recognized as a leading public health concern worldwide, with migraine alone being the second leading cause of disability in women under 50 [1]. Among the headache disorders, migraine, tension-type headache (TTH) and medication overuse headache (MOH) are considered major contributors to the global headache burden [2, 3].

There is a lack of data on the burden of headache disorders from representative population-based studies in Europe. Detailed knowledge of the prevalence, burden, and distribution of headache disorders across demographic groups is essential to plan effective interventions, and to properly target and scale health care services. Until relatively recently, most epidemiological studies on headache disorders have focused on prevalence, while reliable data on headache burden were lacking [2]. The burden of disease is subdivided into *personal burden*, such as detrimental effects on education, family planning or social functioning; and *societal burden*, which includes lost work ability and use of health services. For this reason, the *Lifting The Burden* initiative was launched in collaboration with the World Health Organization (WHO) to quantify and reduce the global burden of headache [4]. In 2014, consensus-based methodological guidelines were published [5] to improve and standardize methods for future cross-sectional studies. These guidelines form the basis for the PopHEAD study.

The HARDSHIP (Headache-Attributed Restriction, Disability, Social Handicap and Impaired Participation) questionnaire has been developed by Lifting the Burden for use in population-based studies to identify the major headache disorders in the general population and to quantify their personal and societal burden [6]. It has since been used in studies in more than twenty countries, including 14 non-EU countries [7–12], all substantially smaller than PopHEAD, but mostly with high response rates and of good quality. European data on headache burden was collected in 2008 as part of the Eurolight project [13], which used a predecessor [14, 15] to the HARDSHIP questionnaire to collect data in 10 EU countries with a total of 9,269 respondents. The EUROLIGHT questionnaire was validated in 5 European languages, including linguistic and a face-content validation, showing good internal consistency. It is considered a reliable and valid instrument to evaluate the burden of headache disorders [15].

A major limitation of the Eurolight project was that only Lithuania conducted a truly population-based survey [13], where 573 participants were included through unannounced home visits [16]. As defined by the Eurolight group, a truly population-based study should have random sampling in a representative part of the general population [14]. The studies in Germany, Italy, Luxembourg and a sub-sample in the Netherlands also had designs that were close to being population-based, but participation rates were low or not reported [17]. In the other countries, various compromises were made, including recruitment through patient organizations, limiting the generalizability of the Eurolight results [17]. Furthermore, the diagnostic questions used in the Eurolight study were not validated in the respective study settings.

Here we aim to describe the methods and validation of questionnaire-based diagnoses in the PopHEAD study, a population-based study on the prevalence and burden of headache disorders using the HARDSHIP questionnaire translated into Norwegian. Advantages of the PopHEAD study over previous European studies include a general population setting, validation of the diagnostic instrument, and the possibility of linkage to retrospective and prospective participant-specific information from national health and prescription registers.

## Methods and materials

### Overview

The PopHEAD Study is a population-based cross-sectional study in which a random sample of 28,753 adults in the Norwegian county of Vestfold and Telemark was invited to participate in a digital questionnaire survey.

### Ethics

Participation was based on informed consent, and the study was approved by the Regional Committee for Medical and Health Research Ethics (REK #355800).

### Study population and recruitment

**Fig. 1:**
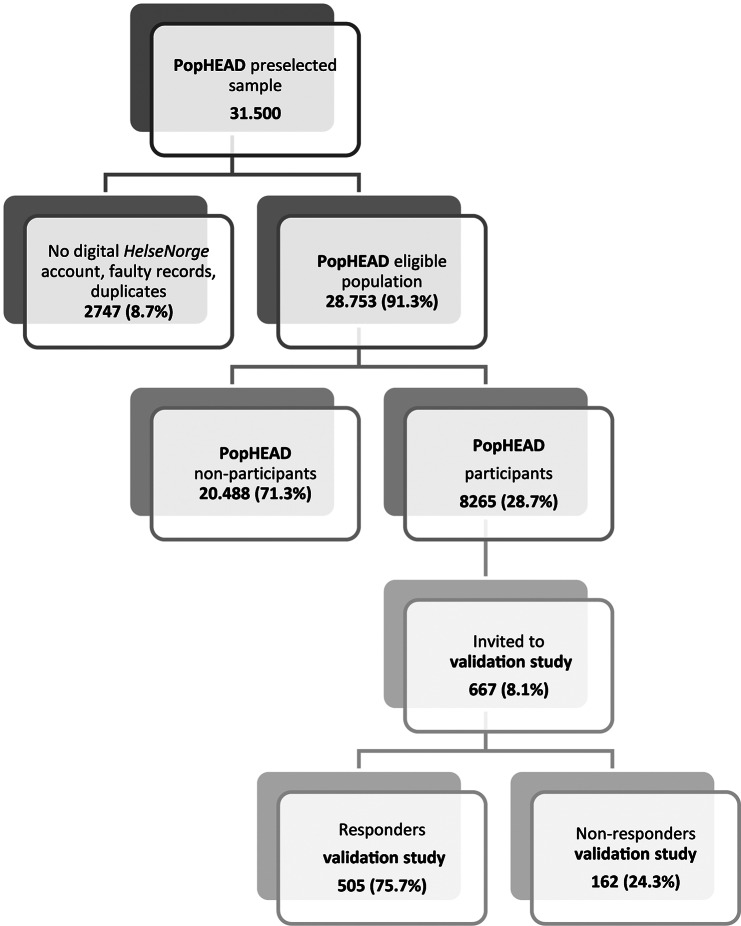
Diagram of the invited population and participation in the PopHEAD study and subsequent validation study

The Vestfold and Telemark County is fairly representative of the general Norwegian population regarding age, sex and household income [18]. A random sample of 31,500 adults (18 to 70 years) living in the county were drawn from the National Population Register, in which all Norwegian citizens are registered. After removing duplicate or incorrect records (*n* = 397), and individuals that did not have an account with the national digital health portal HelseNorge (*n* = 2,350), a total of 28,753 individuals were eligible for participation. The age range of 18–70 years was chosen because headache disorders occur frequently in this age span, and these are the productive years (Fig. 1).

An invitation was sent digitally to each participant via HelseNorge, the official digital communication channel between the health services and the residents of Norway. HelseNorge was accessed via the eFORSK system, which is operated by the Central Norway Regional Health Authority. Invitees were notified by SMS and could access a digital consent form and the questionnaire via the University of Oslo’s secure solution *Nettskjema*. Reminders were sent on days 9 and 29. The reminders linked to a shorter version of the questionnaire, described below, to secure as many respondents as possible. The survey was open for 8 months, but 95% of respondents answered within 4 weeks.

### Study instrument

We translated the full English version of the HARDSHIP questionnaire [6] into Norwegian. The translation process included forward and backward translation with native and bilingual translators using Lifting The Burden’s translation protocol for hybrid documents, which is described in detail elsewhere [19]. The questionnaire has a modular structure consisting of four parts: (i) demographic and socioeconomic questions, (ii) screening questions on lifetime headache (“Have you ever had a headache in your lifetime?”) and headache the past year (“Have you had a headache during the last 12 months?”), and, for those answering “yes” to both screening questions, (iii) diagnostic headache questions, and (iv) burden questions (days absent from work [absenteeism], days with reduced ability to work [presenteeism], and impact on education, career opportunities, income, social commitments, and/or personal relationships). Before conducting the study, we tested the questionnaire in two rounds on 55 volunteers recruited through written notices and verbal information in larger gatherings. Based on their feedback, we made minor changes to improve relevance, comprehensibility, and to ensure the technical solution worked well. The original HARDSHIP questionnaire consists of 101 items. In the PopHEAD questionnaire we did not include the section on interictal burden (4 items), “willingness to pay” (8 items) and anthropometric measures (3 items), but added a follow-up question for those who reported being on sick leave as to whether this was due to their headache disorder (1 item), and a follow-up question on the subjective effectiveness of headache medication (1 item), resulting in a questionnaire comprising 88 items. In the shortened version used for those who needed a reminder, we removed the questions on yesterday’s headache (12 items), the burden on the partner and children (4 items), and questions on quality of life (12 items), reducing the questionnaire from 84 to 60 items. The diagnostic questions were identical in the full and shortened versions of the questionnaire.

### Diagnostic algorithm

The HARDSHIP diagnostic algorithm for migraine and tension-type headache (TTH) has been described in detail previously [19], and applies modified criteria of the International Classification of Headache Disorders (ICHD) version 3 [21]. The headache designated by the participant as the most bothersome was diagnosed in a hierarchical order starting with definite migraine (dMIG), followed by definite TTH (dTTH), probable migraine (pMIG), probable TTH (pTTH) and lastly undetermined headache. Each participant was assigned only one of these diagnoses, which were considered mutually exclusive. As in previous HARDSHIP studies [20, 22–24], we defined migraine as dMIG + pMIG and TTH as dTTH + pTTH. We also validated dMIG and dTTH, henceforth labeled “migraine (strict)”, and “TTH (strict)”, respectively. MOH was defined as headache ≥ 15 days/month and medication overuse during the last 30 days (≥ 10 days/month for triptans, opioids or combination analgesics, or ≥ 15 days/month for simple analgesics). In contrast to previous HARDSHIP studies, MOH and a primary headache (migraine or TTH) were not considered mutually exclusive. In addition to these overall diagnoses specified by the modified HARDSHIP algorithm, we classified participants according to headache subcategories defined in the ICHD-3. Migraine was divided into chronic migraine (participants with migraine who reported ≥ 15 headache days and ≥ 8 migraine days per month) and episodic migraine (participants with migraine who did not fulfill the criteria for chronic migraine). TTH was subcategorized by frequency to fulfill the diagnostic criteria of infrequent (< 12/year), frequent (1–14/month) or chronic (≥ 15 days/month) TTH. The HARDSHIP questionnaire does not include questions on migraine aura and is therefore not designed to classify migraine with and without aura.

### Validation of the questionnaire-based headache diagnoses

To validate the questionnaire-based headache diagnoses, we contacted a random subsample of respondents by telephone within 4 weeks of submitting the survey. Calls were made until our predetermined target of 500 respondents was reached. A total of 667 people were contacted, of whom 505 responded. To ensure balanced participation in terms of age, every second call was made to respondents aged ≥ 50 and < 50 years. We ensured that at least every third respondent was male. Two headache neurologists, each with over 10 years of experience in the treatment of headache patients and blinded to questionnaire responses, conducted all interviews using a semi-structured interview. They diagnosed the most bothersome headache, and up to two additional headache types, using ICHD-3 criteria [21]. For respondents who reported migraine as their most bothersome headache and additionally had TTH, we conducted a structured interview about the individual and societal burden of their TTH to enable subsequent studies of the hidden burden of TTH when comorbid with migraine. Aspects of the HARDSHIP questionnaire other than headache diagnoses were not validated. The ICHD-3 classification does not include a diagnosis for “Probable chronic migraine”. For the purposes of the validation study, participants who were diagnosed with ICHD-3 1.5 Probable migraine who had > 15 headache days and > 8 migraine-like days per month, were classified as having chronic migraine.

### Statistical analysis

Demographic variables were compared between responders and non-responders in the overall study and the validation study using the chi-square test (binary variables) or the t-test for independent samples (continuous variables). For the validation analysis, sensitivity, specificity and Cohen’s kappa (κ) with 95% confidence interval (CI) were calculated. We considered κ values = 0 to indicate no agreement, 0.01–0.20 none to slight, 0.21–0.40 fair, 0.41–0.60 moderate, 0.61–0.80 substantial, and 0.81-1.00 almost perfect agreement) [25]. All analyzes were performed using Stata Statistical Software v17.0 (StataCorp, College Station, TX: StataCorp LLC).

## Results

### Study population and participation characteristics

Of the 28,753 people invited, 8,265 (3,344 men and 4,921 women) answered the PopHEAD questionnaire, corresponding to a participation rate of 28.7%. Of these, 2871 responded after the first reminder, and therefore used the simplified version of the questionnaire.

Headache in the previous year was reported by 89.7% of the women (4,414) and 77.0% of the men (2,508). The demographic factors of participants and non-participants were compared using data from the National Population Register on age, sex, marital status, and nationality. Participants were more likely to be older (mean age 47.3, SD 13.9 in participants; 42.7, SD 14.6 in non-participants; p-value < 0.001), female (59.5% vs. 46.3%, p-value < 0.001), married (48.6 vs. 37.3, p-value < 0.001) and of Scandinavian origin (96.3% vs. 89.1%, p-value < 0.001).

Of the 667 people selected for the validation study, 505 (75.7%) responded, while 162 (24.3%) could not be reached, did not speak Norwegian or did not wish to participate. The mean interval between completing the questionnaire and the validation interview was 19.0 days (SD 7.6 days, range 2–28 days). Compared to the non-responders, the responders in the validation study were more often male (33.7% vs. 21%) as a result of the chosen selection procedure, but the groups did not differ in terms of age, habitation (urban/rural) or marital status (Table 1).

**Table 1:** Responders and non-responders in the validation study

	Responders (505/667)	Non-responders (162/667)	*P* value
Women (%)	335 (66.3)	128 (79.0)	0.002
Mean age (SD)	41.9 (12.4)	40.1 (11.6)	0.10
Urban residence (%)	410 (81.2)	135 (83.3)	0.57
Living with partner (%)	353 (69.9)	112 (69.1)	0.88
Headache previous year (%)	448 (88.7)	147 (90.7)	0.47

SD = Standard deviation

### Validation of questionnaire-based diagnoses

Table 2 shows the sensitivity, specificity and kappa statistics for each of the questionnaire-based headache diagnoses compared to ICHD-3 diagnoses made by a headache neurologist.

For the diagnoses in the HARDSHIP algorithm, we found substantial agreement for migraine (sensitivity 77%, specificity 85%, κ 0.61), MOH (sensitivity 0.58%, specificity 0.99%, κ 0.63) and headache previous year (sensitivity 97%, specificity 73%, κ 0.72), and moderate agreement for TTH (sensitivity 77%, specificity 74%, κ 0.51). For the ICHD headache subcategories, there was moderate agreement for episodic migraine, chronic migraine, and infrequent episodic TTH, and fair agreement for chronic TTH, and frequent episodic TTH.

**Table 2:** Sensitivity, specificity and kappa value of questionnaire-based headache diagnosis

	Sensitivity	Specificity	Kappa (95% CI)
	n (%)	n (%)	
Migraine	134/174 (77)	281/331 (85)	0.61 (0.52–0.70)
Migraine (strict)	68/174 (39)	318/331 (96)	0.40 (0.32–0.48)
TTH	188/244 (77)	193/261 (74)	0.51 (0.42–0.60)
TTH (strict)	161/244 (66)	215/261 (82)	0.49 (0.40–0.58)
Headache previous year	431/443 (97)	45/62 (73)	0.72 (0.63–0.80)
Medication-overuse headache	21/36 (58)	462/469 (99)	0.63 (0.60–0.66)
Episodic migraine	107/144 (74)	314/361 (87)	0.60 (0.51–0.69)
Chronic migraine	14/30 (47)	459/475 (97)	0.43 (0.34–0.52)
Infreq. episodic TTH	59/100 (59)	347/405 (86)	0.42 (0.33–0.51)
Freq. episodic TTH	62/118 (53)	322/387 (83)	0.35 (0.26–0.44)
Chronic TTH	5/26 (19)	472/479 (99)	0.24 (0.16–0.32)

Migraine = HARDSHIP diagnosis of definite or probable migraine. Migraine (strict) = HARDSHIP diagnosis of definite migraine. TTH = HARDSHIP diagnoses of definite or probable tension-type headache. TTH (strict) = HARDSHIP diagnosis of definite tension-type headache. Infreq. episodic TTH = Infrequent episodic tension-type headache. Freq. episodic TTH = Frequent episodic tension-type headache. Chronic TTH = Chronic tension-type headache. CI = Confidence interval

## Discussion

We conducted a digital survey of a random sample of 28,753 individuals from the general population, of whom 8,265 responded, making PopHEAD one of the largest population-based studies on headache burden conducted to date.

The HARDSHIP questionnaire has been translated into more than 20 languages and used in numerous population-based studies [7]. We translated the questionnaire into Norwegian using the Lifting The Burden translation protocol for hybrid documents [19] according to the guidelines of the Global Campaign against Headache [5], making results comparable with previous and future studies.

The questionnaire-based diagnoses were validated in a random subsample of 505 participants by a diagnostic telephone interview with a headache neurologist blinded to the questionnaire responses. The diagnostic interview was conducted in close temporal relation to the survey (2–28, mean 19 days), which is important as the reliability of the validation results decreases rapidly over time [26]. While face-to-face interviews remain the gold standard for the diagnosis of headache, and the absence of a clinical examination can lead to misdiagnosis in some cases, telephone consultations have also become increasingly common in clinical practice, particularly for non-acute headaches following the COVID-19 pandemic [27], and is considered satisfactory for most neurologists and patients.

The agreement was substantial for migraine and MOH and moderate for TTH. The HARDSHIP diagnoses for migraine and TTH have been validated previously in one Russian [23] and five non-European populations (North India, South India, Pakistan, Zambia and China) [20, 22, 24, 28, 29], but not yet in a Western European population. The earlier validation studies were smaller than in PopHEAD (180–399 participants) but showed similar agreement for TTH (κ = 0.39–0.59 vs. 0.51 in PopHEAD). For migraine, agreement was slightly higher in PopHEAD (κ = 0.61) than in the validation studies in India (κ = 0.46 and 0.43), Pakistan (κ = 0.56) and Russia (κ = 0.58), but lower than in China (κ = 0.82) [20, 22–24, 28]. Validation was also attempted in Zambia, but significant practical and methodological challenges made the results unreliable and difficult to interpret [29]. The large validation sample in PopHEAD also allowed us to classify and validate subcategories of migraine and TTH defined in the ICHD-3 (chronic and episodic migraine, infrequent and frequent episodic TTH, and chronic TTH). These subcategories showed fair to moderate agreement with the neurologists’ diagnoses and can be used in future studies to investigate the relative burden of these specific headache subcategories.

Compared to non-participants, participants in PopHEAD were more often older, female, married and had Scandinavian citizenship. The proportion of participants reporting headache in the last year (75% for men and 89% for women) was somewhat higher than in the Lithuanian HARDSHIP study (66% for men and 84% for women) [16], suggesting some recruitment bias in favor of headache sufferers in PopHEAD.

Participation rates in health surveys among the general population have declined over time [30]. In a recent Australian study designed to measure response rate and preferred method of reply for a headache survey using HARDSHIP questions, a survey was sent to 20,000 households with the option to reply digitally or on paper. The study achieved a response rate of only 5% [31]. In the 15-year-old Eurolight study, four countries (Germany, Italy, Luxembourg, sub-sample in the Netherlands) used surveys distributed by mail or via the Internet in approximately population-based designs. The participation rate was not reported in the Dutch study, lower in Germany (11.2%) and Italy (14.3%) and similar to Luxembourg (31.1%) when compared to the present study (28.7%) [17]. We applied several strategies to maximize the participation rate in PopHEAD. First, we ran a media campaign across multiple platforms (national and local television, radio and newspapers) the week before the questionnaire was distributed to increase awareness. Second, we sent out two digital reminders. Third, we shortened the length of the questionnaire for those who responded after the first reminder. Fourth, since 97% of Norway’s adult population uses a smartphone daily [32], and the rate is probably even higher in the age group PopHEAD targeted (adults ≤ 70 years), we believe that the fully digital format reduced the barrier for participation. One factor that may have reduced the participation rate was that the form was only available in Norwegian, thus excluding the non-Norwegian speaking part of the population. This is reflected by the lower participation of non-Scandinavian citizens.

Most population-based surveys are subject to recall bias related to the use of retrospective self-reported measures, such as the number of headache days in the last three months or 30 days. Previous studies have shown that estimates obtained from 3-month recall may be too low compared to data free of recall bias [26]. For this reason, the HARDSHIP questionnaire includes additional detailed questions on ‘headache yesterday’ to provide an estimate of 1-day prevalence and burden of headache that is robust to recall error [6]. Ideally, a prospective study in which subjects keep a diary of headache days and medication use would correct this error and minimize recall bias. This is however challenging to conduct in a population-based setting.

Epidemiologic studies on headache disorders typically limit participants to one headache diagnosis, since diagnosing more than one headache type in the same person using questionnaires is challenging. While some studies apply a hierarchy of diagnoses, usually prioritizing migraine over TTH, others, such as the ones using HARDSHIP, focus on the headache that the participant recognizes as the most bothersome. Migraine and TTH are both common and often co-occur, in which case migraine is likely to be designated the most bothersome headache. Both approaches will therefore tend to underestimate the burden of TTH. In our validation study, we collected data on the presence and specific burden of TTH in individuals with migraine as their most bothersome headache, to allow for the estimation of this ‘hidden burden’ of TTH. Furthermore, previous HARDSHIP studies have defined MOH and primary headache (migraine or TTH) as mutually exclusive diagnoses [7–13], whereas we have allowed the simultaneous diagnosis in the same individual. This is according to the ICHD-3 criteria and should lead to more accurate estimates. Previous HARDSHIP studies have also categorized all headaches with a frequency of more than 15 days/month as “other headache ≥ 15 days/month” and omitted these from the primary headache estimates. The difference in classification between previous HARDSHIP studies and PopHEAD is worth noting. It can be argued that the diagnosis of a primary headache regardless of frequency will give more accurate estimates. The purpose of this validation study was, however, not to compare with other similar studies, but instead to demonstrate the validity of the diagnoses in PopHEAD for further, accurate measures of burden estimates.

## Conclusions

The PopHEAD study provides detailed information on the headache-attributable burden in 8,265 participants from the general population. The questionnaire-based diagnoses showed substantial agreement with neurologists’ diagnoses for migraine and MOH and moderate agreement for TTH. The PopHEAD sample will serve as a basis for studies on personal and societal headache burden and fills a geographical knowledge gap for Western Europe, where representative studies are lacking. All individuals living in Norway have a unique personal identification number that will allow linkage of PopHEAD to electronic health records, national health registers and medical quality registers. This linkage will enable retrospective and prospective analyses, and increase the potential of PopHEAD for future research on headache disorders.

## Data Availability

No datasets were generated or analysed during the current study.

## Abbreviations

CI: Confidence interval
CM: Chronic migraine
CTTH: Chronic tension type headache
EM: Episodic migraine
EU: European Union
FETTH: Frequent episodic tension type headahce
GBD: Global burden of disease
HARDSHIP: Headache–Attributed Restriction, Disability, Social Handicap and Impaired Participation
HUNT: Helseundersøkelsen i Nord Trøndelag
ICHD: International classification of headache disorders
IETTH: Infrequent episodic tension type headache
HIS: International headache society
LBT: Lifting The burden
LLC: Limited liability company
REK: Regionale etiske kommite
SD: Standard deviation
SE: Standard error
TSD: Tjeneste for sikker data
TTH: Tension type headache
WHO: World health organization
YLD: Years lived with disability
